# Chemically induced transformation of human dermal fibroblasts to hair‐inducing dermal papilla‐like cells

**DOI:** 10.1111/cpr.12652

**Published:** 2019-07-01

**Authors:** Qian Zhao, Na Li, Huishan Zhang, Xiaohua Lei, Yujing Cao, Guoliang Xia, Enkui Duan, Shuang Liu

**Affiliations:** ^1^ State Key Laboratory of Stem Cell and Reproductive Biology, Institute of Zoology Chinese Academy of Sciences Beijing China; ^2^ State Key Laboratory of Agrobiotechnology, College of Biological Sciences China Agricultural University Beijing China; ^3^ University of Chinese Academy of Sciences Beijing China

To the Editor:

Cell‐based hair follicle regeneration provides an alternative treatment for alopecia.^1^ Dermal papilla (DP) is a cluster of specialized fibroblasts located at the base of the hair follicle (HF) which serves as an instructive niche for hair development, cycle and morphogenesis, and plays an indispensable part in HF reconstitution.^2^ However, the limited number of DP cells and the gradual loss of hair‐inducing capacity in isolated DP cells during long‐term subculture in vitro^3^ make it a big challenge to acquire abundant hair‐inducing DP cells for successful hair reconstruction. In a recent pioneering work, Fan et al^4^ revealed that treatment of adult fibroblasts with cell‐free extract from embryonic skin conferred upon them the competency to regenerate hair follicles. Here, we report a chemical cocktail combined suspension culture strategy to induce the generation of hair‐inducing DP‐like cells from foetal‐derived or adult foreskin‐derived fibroblasts. And these transformed cells prompt and integrate in reconstructed HF in vivo. This simple and practical methodology makes it possible to obtain abundant hair‐inducing cells for hair loss treatment.

Microarray data showed that DP cells and dermal fibroblasts originate from common fibroblast progenitors in the developing embryonic mouse skin and have highly correlated gene expression profiles (96%).^5^, ^6^ Besides, adult dermal fibroblasts can be reprogrammed into a neonatal state with the capacity of inducing ectopic follicle formation.^7^ Based on these, we speculated that transformation of human fibroblasts to HF‐inducing cells might also be realized by applying appropriate signals in vitro. In vivo studies of DP development give us some guidance: epidermal FGF20 and PDGF‐A participate in the regulation of DP formation, as well as Shh^8^, ^9^, ^10^, ^11^; TGF‐β2 and BMP7 secreted by the β‐catenin‐over‐activating epidemic cells direct ectopic HF formation.^7^ Besides, administration of FGF2, BMP2, BIO, and suspension culture in vitro showed maintains hair‐inductive activity of DP cells.^3^, ^12^ Therefore, candidate factors mentioned above (Shh, PDGF, FGF20, TGF‐β2, BMP7, FGF2, BMP2, BIO) and suspension culture were applied in the transformation of human fibroblasts.

Human DP signature genes, including signal transduction components (*Bmp2*, *BMP4*), transcription factors (*Foxo1*, *Lef1*, *Rgs2*, *Sox2*, *Trps1*) and extracellular adhesion gene (*Vcan*),^3^, ^6^ are enriched in DP cells by several folds to a thousand folds compared with human foetal fibroblasts (Figure S1). These signature genes were picked as markers to evaluate the transformation. The efficiencies of different combinations of factors (Figure S1), treatment times and suspension culture (Figure S2) were evaluated, respectively, by DP signature genes. Results showed that treatment with the combination of FGF2, PDGF and BIO for 6 days in adherent culture followed by suspension culture with these three factors for 24 hours (Figure 1A) could be the best transformation inducing strategy which was conducted in both human foetal fibroblasts and adult fibroblasts. After treatment, foetal fibroblasts gradually changed into less flattened morphology (Figure 1B). The results of RT‐PCR, immunohistochemistry and Western blotting showed outstanding up‐regulation of DP signature genes (Figure 1C, Figure S3). Similar results were seen with adult fibroblasts (Figure 1D,E). Despite some minor differences in the gene expression between foetal and adult fibroblasts, it was easy to tell that transformed human fibroblasts acquire some DP characteristics which we called DP‐like cells.

**Figure 1:**
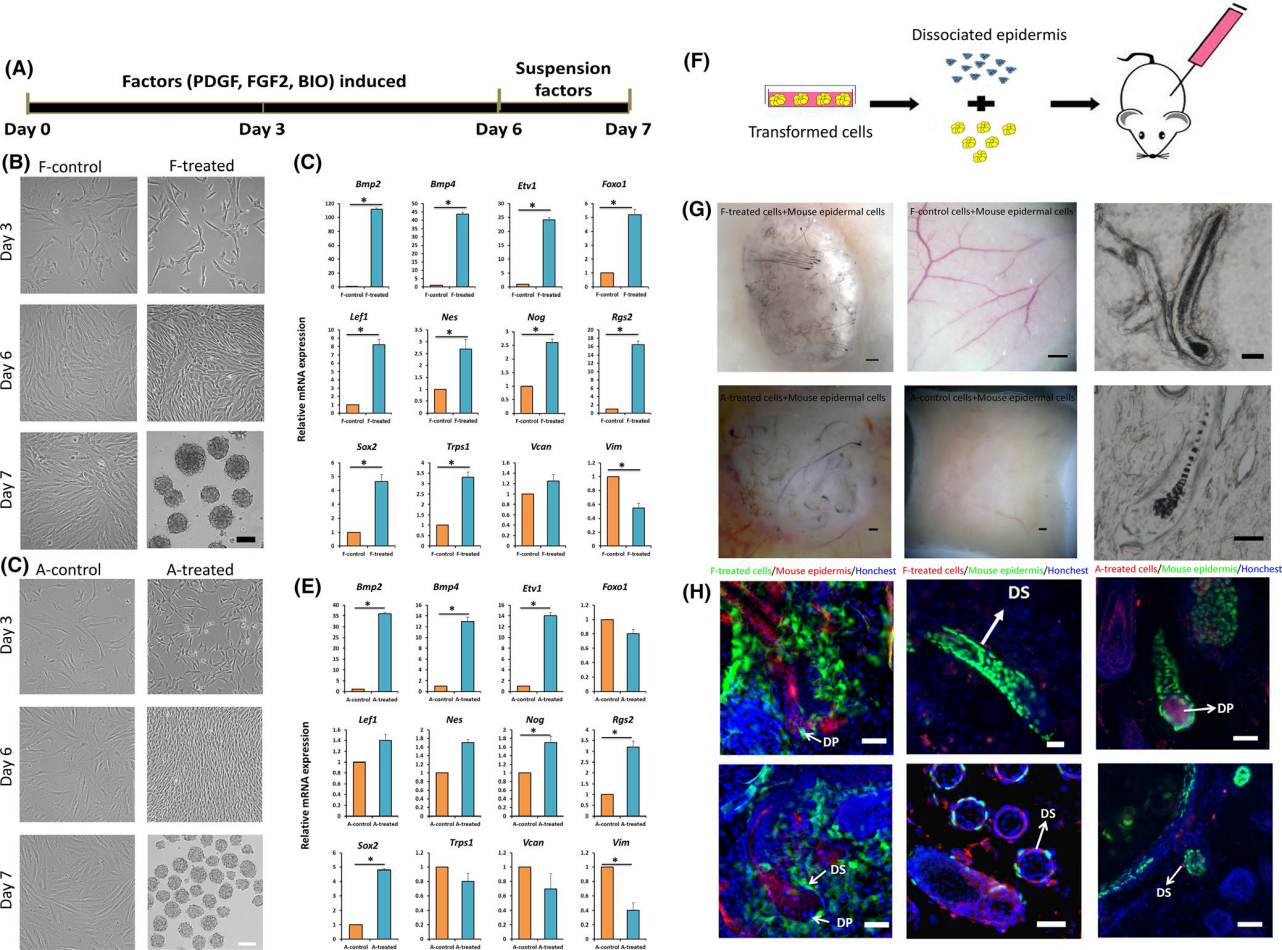
Chemically induced transformation of human fibroblasts to hair‐inducing DP‐like cells. A, Schematic representation of the chemically induced transformation of human fibroblasts to hair‐inducing DP‐like cells. B, Morphology changes of foetal fibroblasts and transformed cells induced by the transformation strategy represented by (A) from foetal fibroblasts. C, qRT‐PCR analysis (mean ± SD; n = 3) of DP signature genes in foetal fibroblasts and transformed cells induced by factors from foetal fibroblasts. D, Morphology changes of adult fibroblasts and transformed cells induced by the transformation strategy represented by (A) from adult fibroblasts. E, qRT‐PCR analysis (mean ± SD; n = 3) of DP signature genes in adult fibroblasts and transformed cells induced by factors from adult fibroblasts. F, Schematic diagram of hair follicle reconstruction. G, Stereo images of skin whole mounts from nude mice transplanted of newborn mouse epidermal cells combined with DP‐like cells (left) or untreated fibroblasts (middle) and the images of reconstructed hair follicle (right). H, Section staining of the reconstructed hair follicles induced with transformed cells from foetal fibroblasts (left and middle) or adult fibroblasts (right). Hoechst labels nuclei in blue. *P < 0.05, scale bar = 100 μm for (B) and (D), 300 μm for (G) and 50 μm for (H)

To access the hair‐inducing capacity of DP‐like cells, “patch assay” was performed as described^13^ (Figure 1F). To track the implanted cells, human fibroblasts were labelled with EGFP before transformation and then implanted into the back of nude mice together with RFP‐expressing neonatal mouse epidermal cells. Three weeks after implantation, we found HF formation in all positive control implanted with newborn mice dermis (Figure S4A), and no hair follicles were observed in control groups with foetal or adult fibroblasts (Figure 1G, Table 1). HF structures were reconstructed in 13 of 20 (65%) nude mice implanted with foetal DP‐like cells and in seven of 10 (70%) nude mice with adult DP‐like cells (Figure 1G, Table 1). RFP+ epidermal cells and EGFP+ dermal cells were observed in the back skin of the mice implanted with DP‐like cells, while only EGFP+ dermal cells were observed in mice implanted with fibroblasts (Figure S4B). Section assay further showed that foetal/adult DP‐like cells integrated in the DP and dermal sheath (DS) which can be recruited to replenish DP of de novo generated hair follicles (Figure 1H).^14^ In addition, human fibroblasts labelled with mCherry were also treated and implanted with K14‐H2B‐GFP neonatal mouse epidermal cells in patch assay. The results further confirmed transformed cells integrated into regenerated hair follicles (Figure 1H) which suggested that DP‐like cells obtained certain hair follicle‐inducing capacity.

**Table 1:** Results of hair follicle formation of different groups in the patch assay

Experimental groups	Epidermal cells^a^	Dermal cells^b^	Number of grafted constructs with hair follicles/total number of grafts (%)
Positive control group	Mouse epidermal cells	Mouse dermal cells	15/15 (100)
Foetal treatment group	Mouse epidermal cells	Foetal DP‐like cells	13/20 (65)
Foetal control group	Mouse epidermal cells	Untreated foetal fibroblasts	0/20 (0)
Adult treatment group	Mouse epidermal cells	Adult DP‐like cells	7/10 (70)
Adult control group	Mouse epidermal cells	Untreated adult fibroblasts	0/10 (0)

a Epidermal cells included mouse epidermal cells.

b Dermal cells included mouse dermal cells, transformed foetal/adult DP‐like cells and foetal/adult fibroblasts.

The differences of DP signature gene expression levels between foetal and adult DP‐like cells were obvious, which were consistent with previous studies that foetal fibroblasts are more sensitive than adult fibroblasts to the change in the culture condition.^15^ Nevertheless, it is remarkable that human fibroblasts isolated from hairless foreskin without hair‐inducing capacity could be transformed into hair follicle‐inducing cells in vitro and induce new HF formation in vivo. Intriguingly, we found that in the control group implanted with fibroblasts, implanted epidermal cells were all gone after 3 weeks. We speculated that the transformed cells might provide a more suitable niche which could offer essential epithelial‐mesenchyme interaction for the epidermal cells than fibroblasts and finally contribute to the HF formation in vivo. However, HF regeneration was not always observed in the treatment group, and the number of de novo hair follicles was much smaller compared with that in positive control group. Given the fact that the up‐regulation of DP signature genes was moderate in the transformed cells compared with DP cells, it is likely that the portion of transformed cells is relatively small in the entire culture or/and the degree of transformation is relative low. Besides, it might also attribute to the fact that human cells have poorer interaction with mouse epidermal cells than mouse dermal cells.^16^ The previous research reported a cocktail of organ‐specific extracellular proteins (apolipoprotein‐A1, galectin‐1 and lumican) are able to induce HF neogenesis from adult fibroblasts, which were selected by proteomics analysis of embryonic whole‐skin extracts.^4^ However, from the perspective of epithelial‐mesenchymal interactions in DP development in vivo and intrinsic properties maintain in vitro, we revealed another different induction system that three factors of PDGF, FGF2 and BIO composed chemical cocktail along with suspension culture to induce human fibroblasts especially adult foreskin fibroblasts into hair‐inducing DP‐like cells and contribute to HF regeneration. Given the trans‐differentiation efficiency, the induction strategy of factors in two studies or additional other factors might further enhance the efficiency. In conclusion, we demonstrated a simple and feasible way to acquire abundant hair follicle‐inducing donor cells by our chemical cocktail in hair loss treatment with the propagation ability of fibroblasts.

## CONFLICT OF INTEREST

The authors have no financial conflicts of interest.

## AUTHORS' CONTRIBUTIONS

QZ and NL performed the experiments; QZ collected data and prepared the manuscript. HZ, XL, YC and GX assisted in the experiments and analysed the data. QZ, SL and ED designed the study, analysed data and edited the manuscript.
