# Supplementary figures and images for: Chemically induced transformation of human dermal fibroblasts to hair‐inducing dermal papilla‐like cells
Source: Cell Prolif. 2019 Jul 1;52(5):e12652. doi: 10.1111/cpr.12652 (PMC6797507; doi:10.1111/cpr.12652)

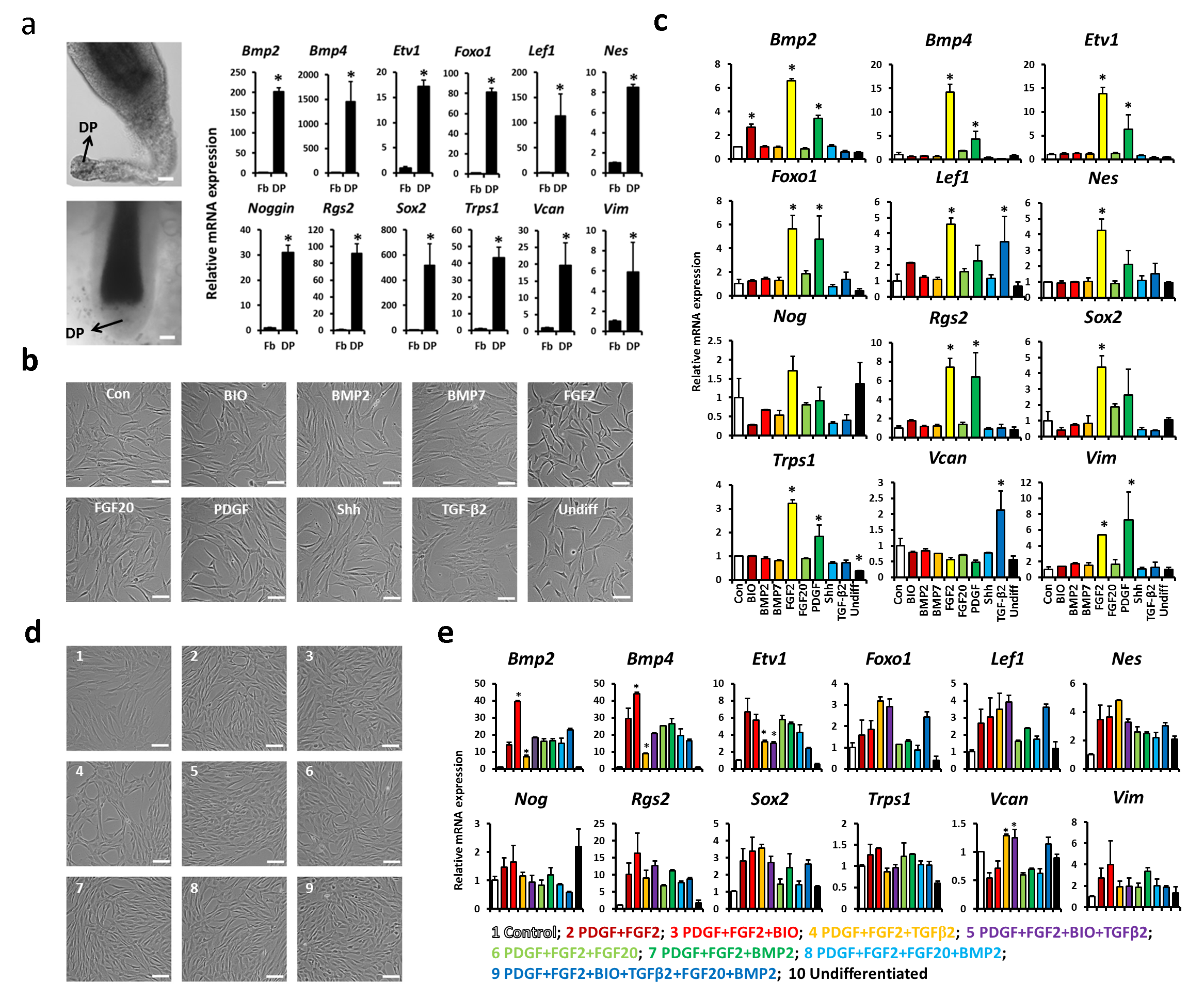

Supplement: Supplementary file 1 [file CPR-52-e12652-s001.tif]

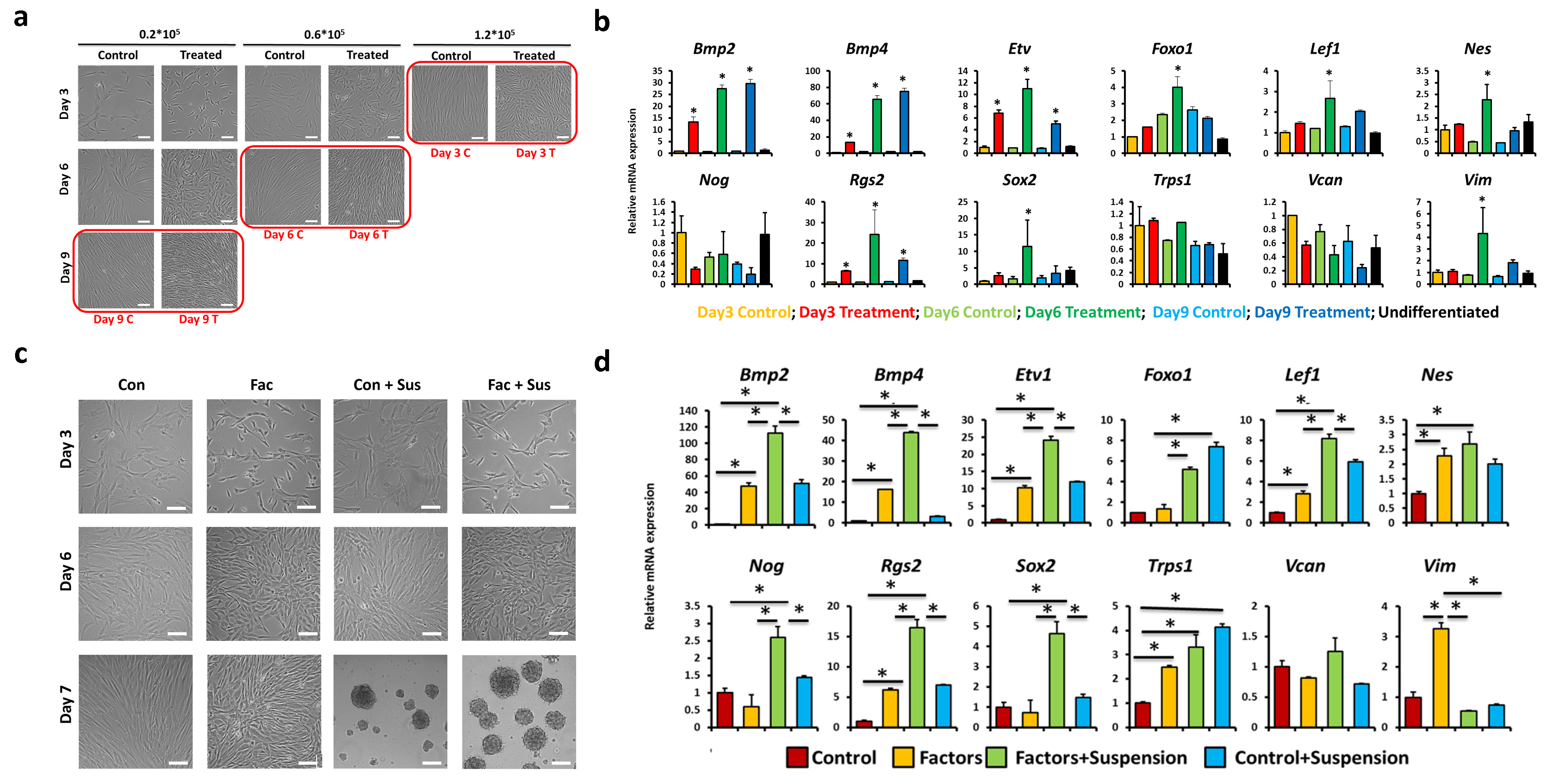

Supplement: Supplementary file 2 [file CPR-52-e12652-s002.tif]

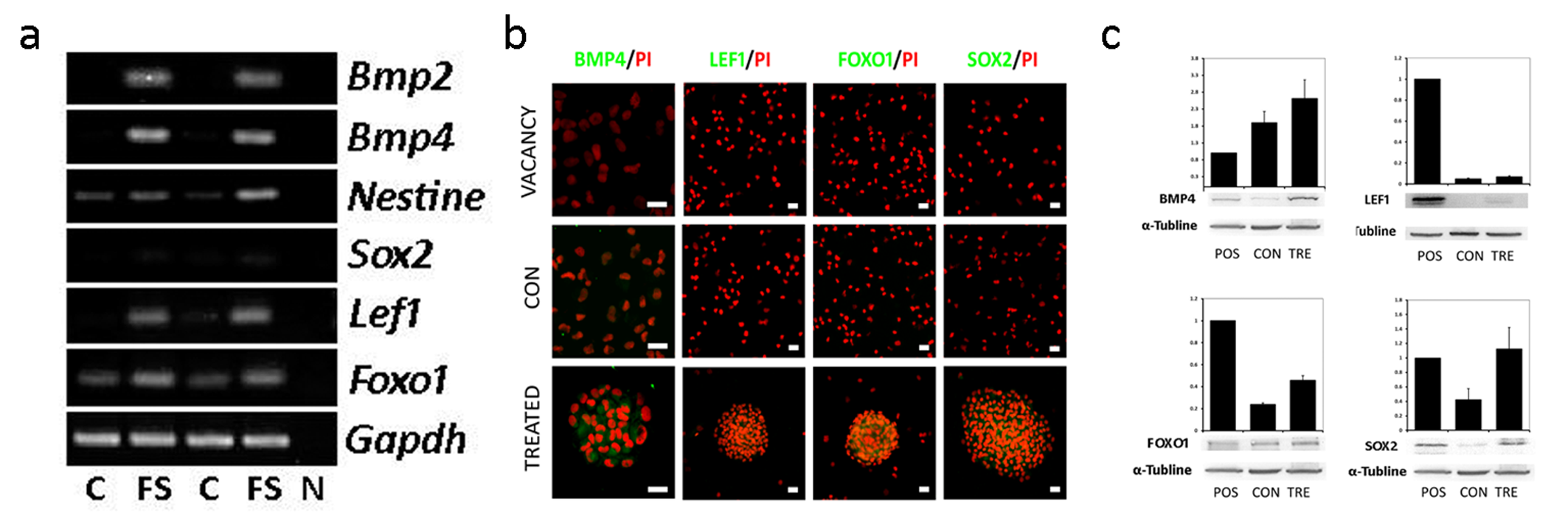

Supplement: Supplementary file 3 [file CPR-52-e12652-s003.tif]

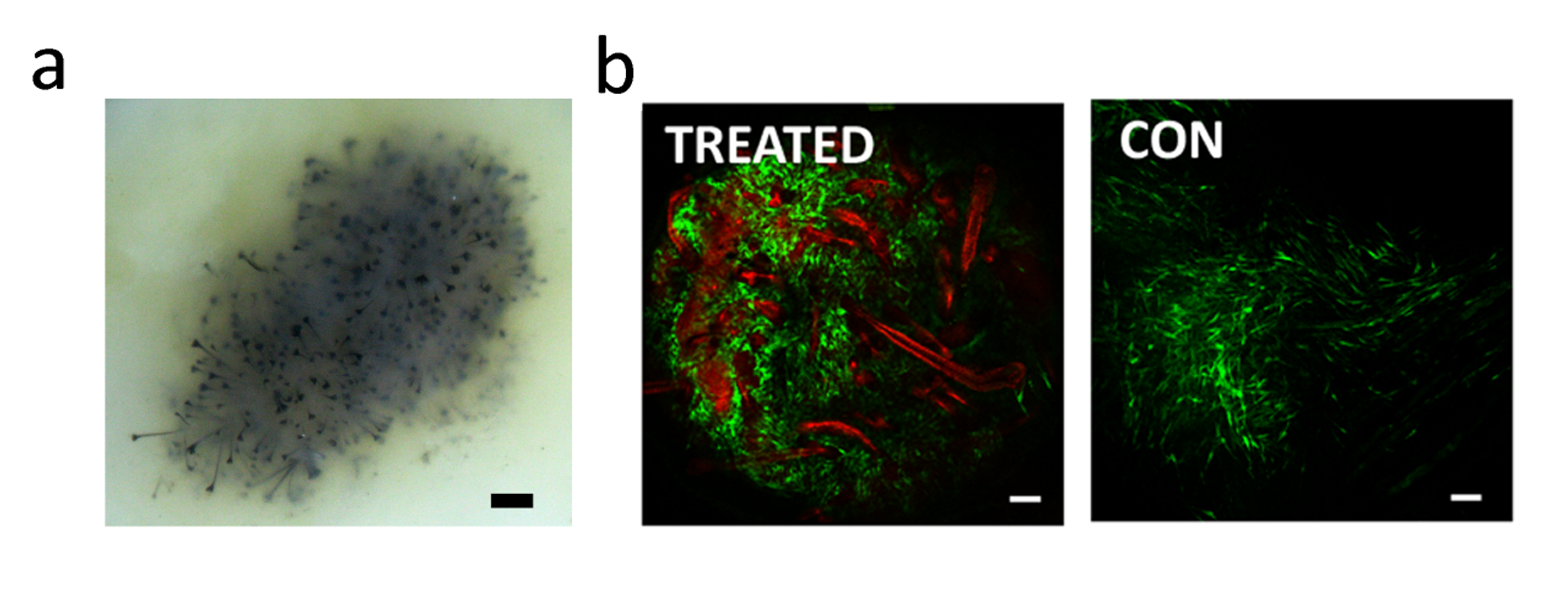

Supplement: Supplementary file 4 [file CPR-52-e12652-s004.tif]
