# Supplementary material for: Chemically induced transformation of human dermal fibroblasts to hair‐inducing dermal papilla‐like cells
Source: Cell Prolif. 2019 Jul 1;52(5):e12652. doi: 10.1111/cpr.12652 (PMC6797507; doi:10.1111/cpr.12652)
Supplement: Supplementary file 5 [file CPR-52-e12652-s005.docx]

**Supplementary Information**

**MATERIALS AND METHODS**

***Human fetal and adult fibroblasts culture***

All experiments were handled according to animal care protocols approved by the Guideline of the Ethics Committee of the Institute of Zoology, Chinese Academy of Science (CAS). Human fetal and adult fibroblasts were isolated from the foreskins of the voluntary circumcisions with informed consents and the method has been described previously^1^. The isolated fibroblasts were expanded for at least ten passages in DMEM (Invitrogen) growth medium supplemented with 10% fetal bovine serum (FBS) (Gibco), 1x penicillin and streptomycin (Sigma) at 37 ℃ with 5% CO_2_. For transformation induction, cells were cultured in the basal transformation DMEM medium with 5% FBS and corresponding factors were added in different experimental groups with fixed concentrations (SHH, 20 ng/mL; BMP2, 20 ng/mL; PDGF, 20 ng/mL; TGF-β2, 20 ng/mL; BMP7, 20 ng/mL; FGF2, 20 ng/mL; BIO, 0.5μM) (all from Peprotech). For suspension culture, cells were suspended and plated in low-attachment petri dishes (Corning) at a density of 4×10^5^ cells/mL and cultured in DMEM medium with 10% FBS and inducing factors. For cell passage, 0.25% trypsin-EDTA (Invitrogen) was used.

***Chemical-induced transformation of human fibroblasts***

To obtain the best transformation-inducing strategy, we evaluated the effects of different combinations of the candidate factors (including Shh, PDGF, FGF20, TGF-β2, BMP7, FGF2, BMP2 and BIO), different treatment durations and the effects of suspension culture. Human DP signature genes, including *Bmp2, BMP4, Foxo1, Lef1, Rgs2, Sox2, Trps1* and *Vcan* which were enriched in DP cells by several folds to a thousand folds compared with human fetal fibroblasts were used as markers to evaluate the transformation.

First, human fetal fibroblasts were treated with each candidate factors respectively for 6 days. Cells treated with FGF2 and PDGF exhibited most drastic up-regulation of most DP enriched genes and corresponding morphology changes (Fig S1b and c). Then we tested the different combinations of factors based on FGF2 and PDGF. The morphology changes of the fibroblasts were observed in all treated groups (Fig S1d). qRT-PCR results showed that the combination of FGF2+PDGF+BIO exhibited the optimum effects on DP signature gene expression, which was regard as the optimal combination of the factors (Fig S1e). After that, we further evaluated the effects of different treatment durations. Results indicated that the 6-day treatment induced the most drastic up-regulation of DP signature genes (Fig.S2a and b). On the basis of above results, we further explored the effects of spherical suspension culture. Since long-term spherical culture of DP cells leads to cell death, the cells were only cultured in suspension for24 hours. The cell morphology changes were presented in Figure S2c and Figure 1b. qRT-PCR assays indicated that suspension culture with factors most drastically improved the expressions of most DP signature genes (Figure S2d, and Figure 1b, c). Finally, the transformation strategy was confirmed as combined treatment of FGF2+PDGF+BIO for six days, and then suspension culture with factors for 24 hours and generated the transformed cells which we called DP like cells.

***Labeling of human fibroblasts with EGFP and mCherry***

Lentivirus production was performed as previously deacribed ^2^. Brifely, the lentiviral vectors for infection were packaged using ViraPowerTM Lentivirus Packaging System (Invitrogen) according to the manufacturer’s instructions. 293FT cells (Invitrogen) at nearly 50% confluence were transfected with target plasmids for the green fluorescent protein (GFP) plasmis and mCherry plasmid which were constructed in pLKO.201 and PLL3.7-EF1 (Invitrogen) respectively and supermix plasmids (Invitrogen, Carlsbad, CA, USA) using the Fugene HD (Roche) transfection reagent. After overnight incubation, the medium was replaced, and after 48 hours and 72 hours, supernatant containing lentivirus was collected and concentrated with ultracentrifuge at 20,000 rpm for 1.5 hours at 4°C.

For infection, 10^5^ human fibroblasts were incubated for 24 hours in growth medium containing concentrated lentivirus and 8 μg/mL polybrene (Sigma). The virus-containing growth medium was replaced with fresh growthmedium after 24 hours and subculture for two passage. EGFP-fibroblasts and mCherry-fibroblasts were enriched by flow cytometry and expanded before transformation.

***Isolation of human dermal papilla***

Human scalp specimens were obtained from surgical patients with informed consents. Intact dermal papilla were manually isolated from scalp follicles by micro-dissected as previously described ^3^. Briefly, small scalp specimens were transected at the dermis-subcutis of hair follicle embeded in the adipose tissue. The fat tissue were pressed carefully with blunt forceps to partially extrude the upper portion of the HFs from the subcutis, and then gripped and extracted the HFs from the subcutis in DMEM. Under bright-field stereoscopic illumination, the connective tissure sheath capsule was transsected at the level of the stalk, thereby “opening up” the proximal capsule just underneath the dermal papilla. And then the gentle pressure imparted by the gripped forceps and to release the dermal papilla from the bulb.

***RNA extraction, reverse transcription and quantitative RT-PCR (qRT-PCR)***

Cells were digested and collected by centrifugation, total RNA was extracted using a Trizol reagent (Promega) according to the manufacturer’s protocol. After the removal of genomic DNA with RQ1 RNase-Free DNase, cDNAs were synthesized with M-MuLV reverse transcriptase (Promega). RT-PCR was performed in a 20 μl reaction volume with Promega PCR Master Mix as described previously, and glyceraldehyde 3-phosphate dehydrogenase (GAPDH) was used for normalization. qRT-PCR was performed using GoTaq qRT-PCR master mix (Promega) in a Roche LightCycler 480 system with a preincubation at 95 °C for 2 min; followed by 35 cycles of amplification at 95 °C for 15 s, 60 °C for 15 s, and 70 °C for 15 s. GAPDH was used for normalization, and the primers used for the qRT-PCR are listed in Table S1.

***Immunofluorescence staining***

Cells were cytospinned and fixed with 4% paraformaldehyde (Sigma), washed in PBS and then permeablized and blocked in PBS with 5% BSA (Sigma) and 0.2% triton-X 100 (Sigma) at 37 ℃ for 1 h. Primary antibodies were applied in PBS with 1% BSA and 0.2% triton-X 100 at 4 ℃ overnight. After washing the slides, appropriate secondary antibodies were applied at 37 ℃ for 1 h. Slides were washed and then propidium iodide (PI) was used for nuclear staining. Primary antibodies used were as follows: rabbit anti-BMP4 (1:100) (Abcam), rabbit anti-LEF1 (1:100) (Abcam), rabbit anti-FOXO1 (1:100) (Abcam), rabbit anti-SOX2 (1:100) (Abcam). The secondary antibodies (1:200) were purchased from ZSGB Biotechnologies (Beijing, China).

***Western blotting***

Total proteins were extracted using RIPA lysis buffer (Beyotime) according to the manufacturer’s instruction. Protein lysates were mixed with 5× SDS sample loading buffer (Beyotime) and heated at 95 ℃ for 5 min. For immune blotting, proteins were resolved by SDS–PAGE and transferred into PVDF membranes (Millipore). After blocked with 5% non-fat milk in TBST (pH 7.4) for 1 hour at room temperature, membranes were incubated with primary antibodies diluted in blocking buffer at 4 ℃ overnight. After incubation with horseradish (HRP)-conjugated secondary antibodies and several washes, membranes were detected with Immobilon Western HRP substrate (Millipore). Primary antibodies used were as follows: rabbit anti-BMP4 (1:1000) (Abcam), rabbit anti-LEF1 (1:1000) (Abcam), rabbit anti-FOXO1 (1:1000) (Abcam), rabbit anti-SOX2 (1:1000) (Abcam), rabbit anti-α-Tubline (1:1000).

***Hair follicle reconstruction***

Engraftments were performed as described previously ^4^. Briefly, back skins of newborn RFP mitochondria and K14-H2B-GFP C57 mice were isolated and washed in PBS, then incubated in 0.02% Dispase (Invitrogen) dissolved in PBS overnight at 4 ℃. Next day, epidermal layers of skins were isolated with forceps, and then cut into pieces and incubated with 0.25% Trpysin-EDTA solution at 37 ℃ for about 10 minutes. Enzyme activity was blocked with FBS. Single cell suspension was obtained by pipetting and filtering through a 40 μm cell strainer. 50 μL epidermal cell suspension (3×10^7^ cells/ml) combined with 50 μL transformed cell suspension (3×10^7^ cells/ml) were transplanted subcutaneously to immunodeficient female mice (strain Athymic Nu/Nu). After 21 days, mice were euthanized and analyzed hair-follicle-inducing characteristic of DP like cells.

**References**

1. Liu S, Wang X, Zhao Q, et al. Senescence of human skin-derived precursors regulated by Akt-FOXO3-p27(KIP(1))/p15(INK(4)b) signaling. *Cell Mol Life Sci.* 2015;72(15):2949-2960.

2. Liu S, Liu S, Wang XY, et al. The PI3K-Akt pathway inhibits senescence and promotes self-renewal of human skin-derived precursors in vitro. *Aging Cell.* 2011;10(4):661-674.

3. Magerl M, Kauser S, Paus R, Tobin DJ. Simple and rapid method to isolate and culture follicular papillae from human scalp hair follicles. *Exp Dermatol.* 2002;11(4):381-385.

4. Morgan BA. The dermal papilla: an instructive niche for epithelial stem and progenitor cells in development and regeneration of the hair follicle. *Cold Spring Harb Perspect Med.* 2014;4(7):a015180.

**Table S1.** **Primer sequences used for RT-PCR**

| **Gene** | **Primers** |
| --- | --- |
| *Gapdh* | Forward: TGTTGCCATCAATGACCCCTT  Reverse: CTCCACGACGTACTCAGCG |
| *Bmp2* | Forward: TGCTTCTTAGACGGACTGCG  Reverse: AGCAACGCTAGAAGACAGC |
| *Bmp4* | Forward: CACTGGTCTTGAGTATCCTG  Reverse: ACGAAAAGCAGAGTTTTCAC |
| *Etv1* | Forward: CTATGGAGAAAAGTGCCTGT  Reverse: TTGAGTTTGGAGATGCATGA |
| *Foxo1* | Forward: ATCTACGAGTGGATGGTCAA  Reverse: AATGAACTTGCTGTGTAGGG |
| *Lef1* | Forward: GAATTAGCACGGAAAGAAAGA  Reverse: ACCTGTACCTGATGCAGATT |
| *Nestin* | Forward: CAACAGCGACGGAGGTCTC  Reverse: GCCTCTACGCTCTCTTCTTTGA |
| *Noggin* | Forward: CTGGTGGACCTCATCGAACA  Reverse: CGTCTCGTTCAGATCCTTTTCCT |
| *Rgs2* | Forward: CTGTCCTCAAAAGCAAGGAA  Reverse: CCTTTTCTGGGCAGTTGTAA |
| *SOX2* | Forward: TGCGAGCGCTGCACAT  Reverse: TTCTTCATGAGCGTCTTGGTTTT |
| *Trps1* | Forward: AAGAAGAGATTTCCCGACAC  Reverse: CTGTAGTGATGTCCTGTTCC |
| *Vcan* | Forward: CTTCCTATGTGACCCGC  Reverse: CTTCCTATGTGACCCGC |
| *Vim* | Forward: TGCCGTTGAAGCTGCTAACTA  Reverse: CCAGAGGGAGTGAATCCAGATTA |
